# Capillary leakage with inflammation and surgery

**DOI:** 10.1186/2197-425X-3-S1-A286

**Published:** 2015-10-01

**Authors:** A Komáromi, U Estenberg, J Wernerman, O Rooyackers, Å Norberg

**Affiliations:** Karolinska University Hospital Huddinge, Anaesthesia and Intensive Care, Stockholm, Sweden; Karolinska University Hospital Huddinge, Nuclear Medicine, Stockholm, Sweden; Karolinska Institutet, CLINTEC, Anaesthesia, Stockholm Sweden

## Introduction

The patency of the capillary wall is compromised in inflammation, stress, and fluid overload resulting in extravasation of water and macromolecules [1]. Reliable measurement of capillary leakage is an important tool in the pursuit of treatment effects.

## Objectives

To study plasma volume (PV) and transcapillary escape rate of albumin (TER) in relation to acute inflammation and surgical stress.

## Methods

Healthy volunteers (group A; n = 10), patients with acute abdominal inflammation and plasma C-reactive protein > 100 mg/L (group B; n = 10), and surgical patients during the reconstructive phase of pancreatic resection (group C; n = 10) were investigated. PV and TER were measured by ^125^I-labeled human serum albumin (^125^I-HSA). Groups were compared by one-way analysis of variance.

## Results

Thirty subjects 57 ± 9 years were recruited. TER was 4.5 ± 1.3, 6.1 ± 1.5 and 9.6 ± 5.2 % per hour in groups A-C, respectively, (p = 0.006). Mean PV was 111 ± 19, 114 ± 15, and 102 ± 18 % (p = 0.79) of the corresponding anthropometric values [2]. Plasma albumin was 39.3 ± 2.9, 24.7 ± 4.9 and 19.1 ± 5.3 g/L at the start of TER measurement (p < 0.001).

## Conclusions

Capillary leakage assessed by TER was doubled during the later stages of pancreatic resection surgery, compared to the other two groups. While PV was preserved in all 3 groups, plasma albumin was much lower in inflammation and surgery.

## Grant Acknowledgment

Swedish Medical Research Council, and the Country Council of Stockholm
